# Supplementary material for: Gypsum heterogenous nucleation pathways regulated by surface functional groups and hydrophobicity
Source: Nat Commun. 2025 Jan 16;16:713. doi: 10.1038/s41467-025-55993-w (PMC11739488; doi:10.1038/s41467-025-55993-w)
Supplement: Supplementary file 1 — Supplementary Information [file 41467_2025_55993_MOESM1_ESM.pdf]

Supplementary Information

Gypsum heterogenous nucleation pathways regulated by surface functional groups and hydrophobicity

Yan-Fang Guan[1, #], Xiang-Yu Hong[2, #], Vasiliki Karanikola[3], Zhangxin Wang[4], Weiyi Pan[5], Heng-An Wu[2], Feng-Chao Wang[2, *], Han-Qing Yu[1, *], Menachem Elimelech[5, *]

[1]CAS Key Laboratory of Urban Pollutant Conversion, Department of Environmental Engineering, University of Science & Technology of China, Hefei 230026, China.

[2]CAS Key Laboratory of Mechanical Behavior and Design of Materials, Department of Modern Mechanics, University of Science and Technology of China, Hefei, China

[3]Department of Chemical and Environmental Engineering, University of Arizona, Tucson, Arizona, USA.

[4]Institute of Environmental and Ecological Engineering, Guangdong University of Technology, Guangzhou, Guangdong 510006, China.

[5]Department of Civil and Environmental Engineering, Rice University, Houston, TX 77005, USA

Supplementary Note 1.

The surface free energy was calculated using Young's equation, which was developed based on Derjaguin-Landau-Verwey-Overbeek (DLVO) theory[1, 2]. It can be described by Eqs. S1-S3. The surface free energy comprises a Lifshitz–van der Waals component, denoted as γ^{LW}, and a Lewis acid–base component, denoted as γ^{AB} (Eq. S1). The acid–base component γ^{AB} can be further described according to Eq. S2. To determine the values of γ^{LW}, γ^{+}, and γ^{-} for a substrate, three probe liquids (i.e., water, formamide, and bromonaphthalene) with known surface tension components were employed.

$$\gamma^{TOT}=\gamma^{LW}+\gamma^{AB} \quad \text{(S1)}$$

$$\gamma^{AB}=2\sqrt{\gamma^{+}\gamma^{-}} \quad \text{(S2)}$$

$$(1+\cos\theta)\gamma_l^{TOT}=2\left(\sqrt{\gamma_S^{LW}\gamma_l^{LW}}+\sqrt{\gamma_s^{+}\gamma_l^{-}}+\sqrt{\gamma_l^{+}\gamma_s^{-}}\right) \quad \text{(S3)}$$

where γ^{TOT} (mJ m^{-2}) is the total surface free energy, γ^{+} (mJ m^{-2}) represents the capability of electron acceptance, γ^{-} (mJ m^{-2}) is the capability of electron-donating, and θ (°) is the contact angle obtained by three probe liquids mentioned above. The subscript s refers to substrate and l refers to liquid for probing the substrate.

Supplementary Note 2.

In classical nucleation theory, interfacial free energy (γ) signifies the collective energy of the crystal-liquid-substrate system. The net interfacial free energy, γ, is a composite term containing three contributions: the crystal-liquid (γ_{CL}), crystal-substrate (γ_{SC}), and substrate-liquid (γ_{SL}). It can be described by Eq. S4:

$$\gamma=\gamma_{CL}+h(\gamma_{SC}-\gamma_{SL}) \quad \text{(S4)}$$

where h is a nucleus shape factor (for example, h = 1/2 for a hemisphere).

Traditionally, γ_{CL} has been assumed to be constant. Thus, the higher values of γ are associated with large γ_{SC}, small γ_{SL}, or some combination of both. Unfortunately, γ_{SL} is difficult to measure.

Surface energy is typically determined by the contact angles of pure liquids with established surface tension properties on a specific solid surface. Hence, the surface free energy (γ_S) of a solid is described as the change in total surface free energy per unit area, under constant temperature, pressure, and moles. Essentially, it represents the interaction energy between the substrate and the air. Consequently, it is crucial to note that the surface energy cannot merely substitute for γ_{SL} in interfacial free energy.

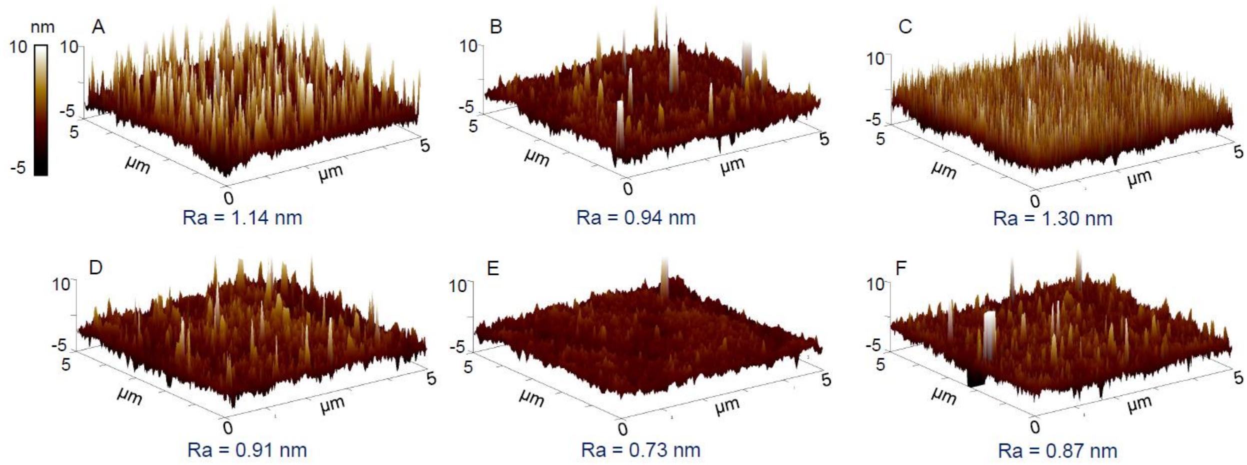

Supplementary Fig. 1 | Representative AFM images for surfaces terminated with (A) −OH, (B) −COOH, (C) $-NH_2$, (D) $-CH_3$, (E) $-SO_3$, and (F) hybrid of NH_2 and COOH groups. The scale bar, shown on the left of Fig. S1A, for all images is 15 nm.

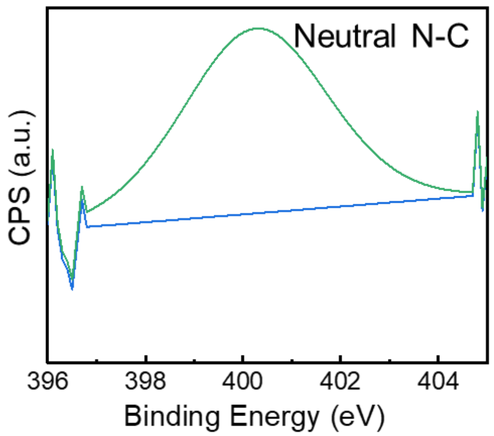

Supplementary Fig. 2 | Deconvolution of N_{1s} spectrum of self-assembled monolayers terminated with $-NH_2$ and $-COOH$ groups.

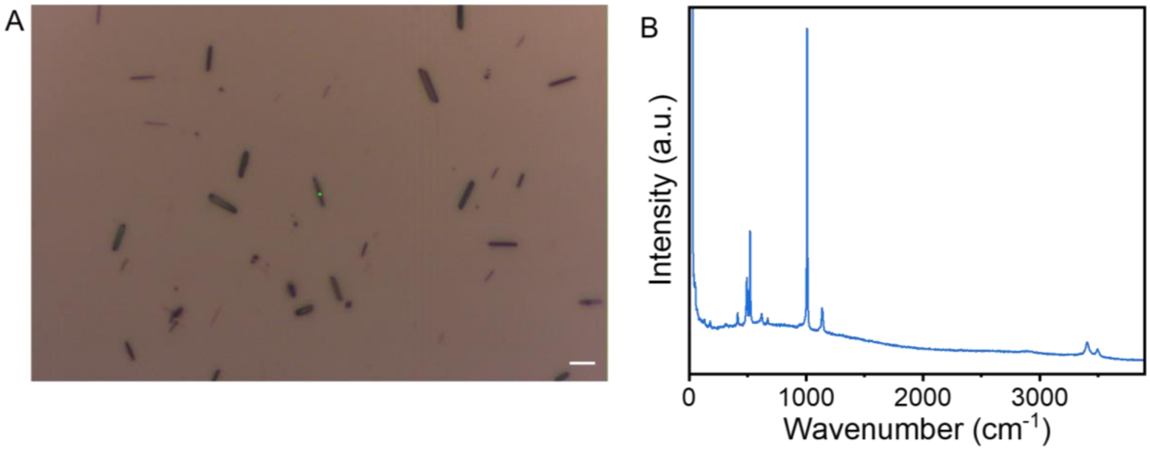

Supplementary Fig. 3 | Confocal micro-Raman spectroscopic characterization. Confocal (A) bright field microscopy image (108 × 68 μm) and corresponding (B) Raman spectrum collected from gypsum nucleated on a –COOH functionalized surface. The Raman spectrum was gathered from the green dot shown in Supplementary Fig. 3A. Peaks at 520, 1004, 1137, 3406, and 3491 cm^{-1} were attributed to the characteristic peaks of gypsum (listed in Supplementary Table 2). Confocal micro-Raman spectroscopic characterization was conducted with a He-Ne 532 nm excitation laser (0.544 mW), a 600 lines/mm grating, and an Olympus LMPlan FLN 50 × objective (N.A. 0.50). To obtain the bright field microscopy image and Raman spectrum, –COOH functionalized surface with gypsum was obtained at the end of nucleation experiments, followed by drying at room temperature. Scale bar = 4 μm.

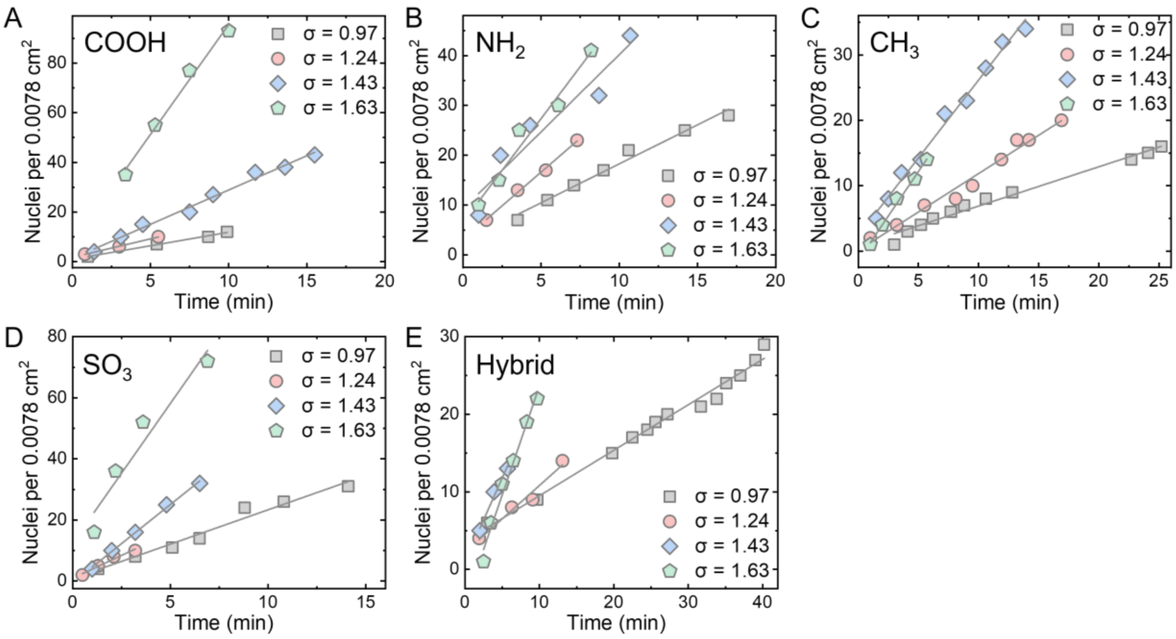

Supplementary Fig. 4 | The number of gypsum crystallites on the surfaces terminated with (A) −COOH, (B) −NH_2, (C) −CH_3, (D) −SO_3, and (E) hybrid of NH_2 and COOH groups increases linearly with time during early stage of each experiment. The slops of the plotted lines for each concentration quantify steady-state nucleation rates.

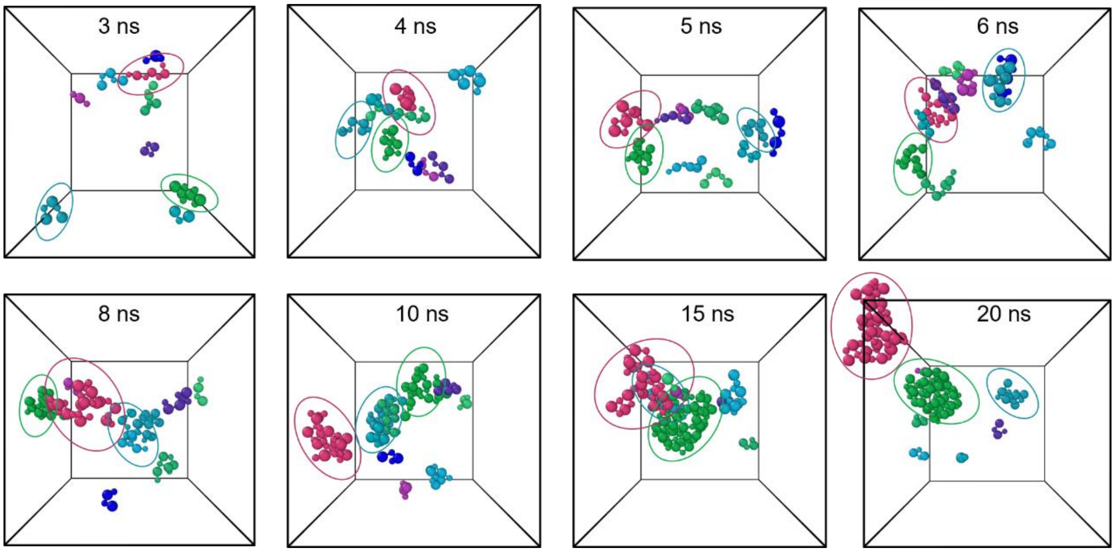

Supplementary Fig. 5 | The dynamic evolution of $CaSO_4$ cluster with time on $–CH_3$ surface. The clusters circled by red, green and blue are the top three largest pre-nucleation $CaSO_4$ clusters. Only the top 10 largest $CaSO_4$ clusters are displayed while the rest are hidden.

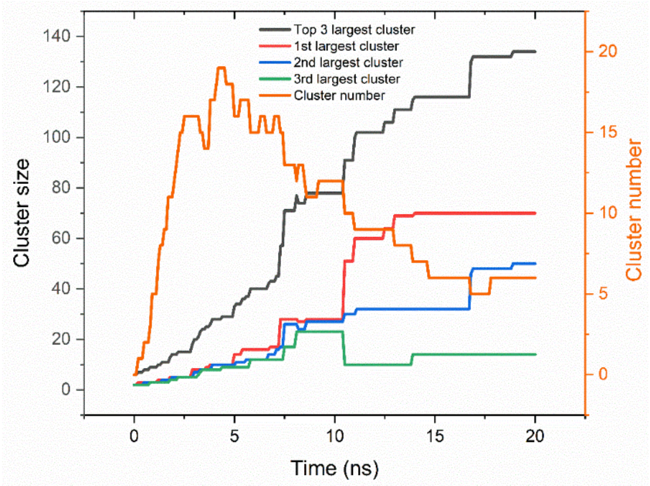

Supplementary Fig. 6 | The time evolution of cluster number and top three largest pre-nucleation $CaSO_4$ clusters on -CH_3 surface.

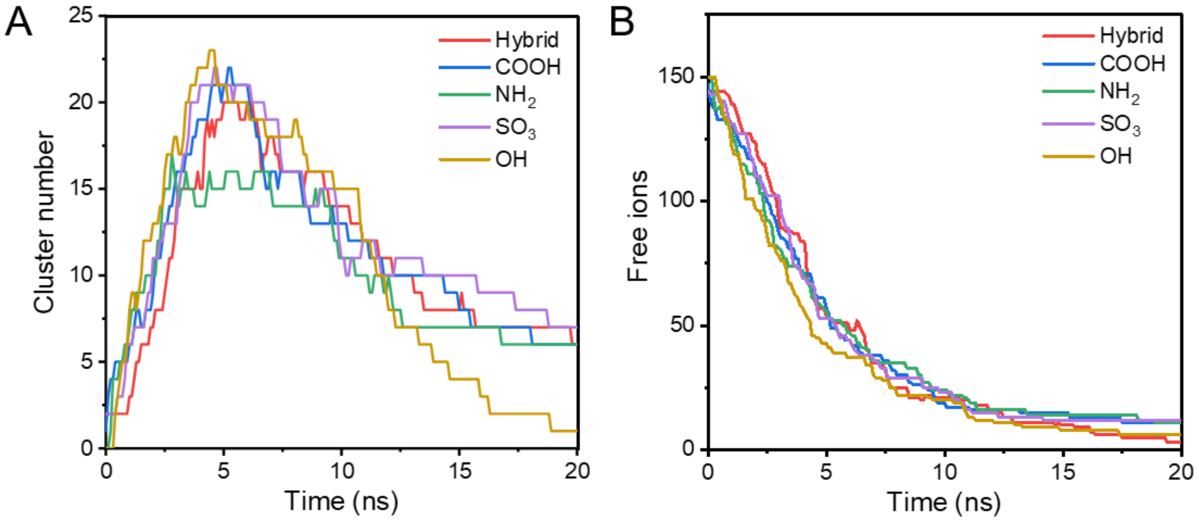

Supplementary Fig. 7 | The evolution of (A) cluster number and (B) free ions as a function of time on surfaces terminated with −COOH, $-NH_2$, −OH, $-SO_3$, and hybrid of NH_2 and COOH groups.

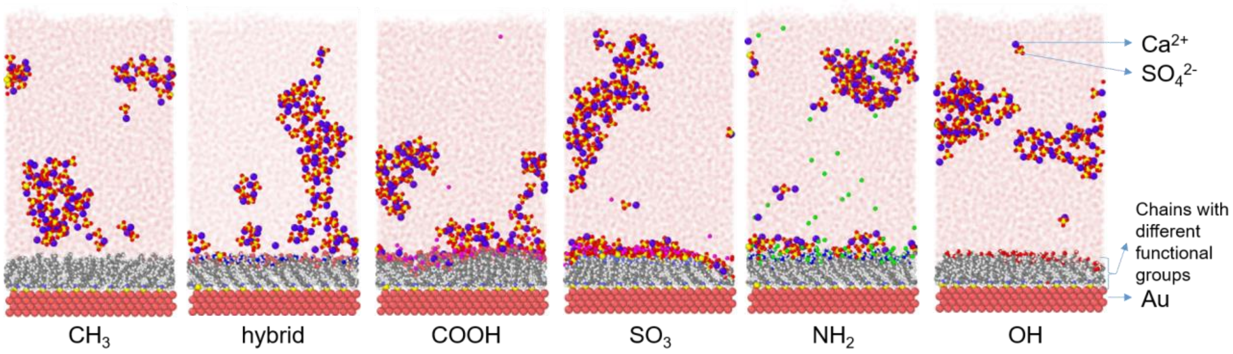

Supplementary Fig. 8 | Simulation snapshots of calcium and sulfate ions distribution on different surfaces at the simulation time of 20 ns. Considering the acidic and basic functional groups can be ionized in the aqueous solution, the ionized forms of hybrid, −COOH, and $-NH_2$ groups were adopted and additional sodium and chloride ions were supplemented to make the system charge neutral.

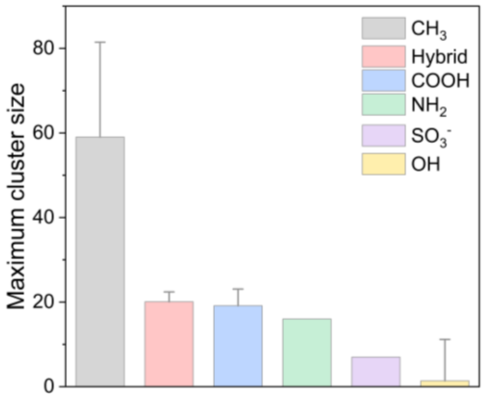

Supplementary Fig. 9 | The maximum cluster size near the surfaces (i.e., the distance between the center of mass (COM) of cluster minus the cluster radius and the surface groups is less than 15 Å) terminated with $-CH_3$, $-COOH$, $-NH_2$, $-SO_3$, $-OH$, and hybrid of NH_2 and COOH groups. Error bars are derived from the standard deviation of the average values calculated over the last 5 ns.

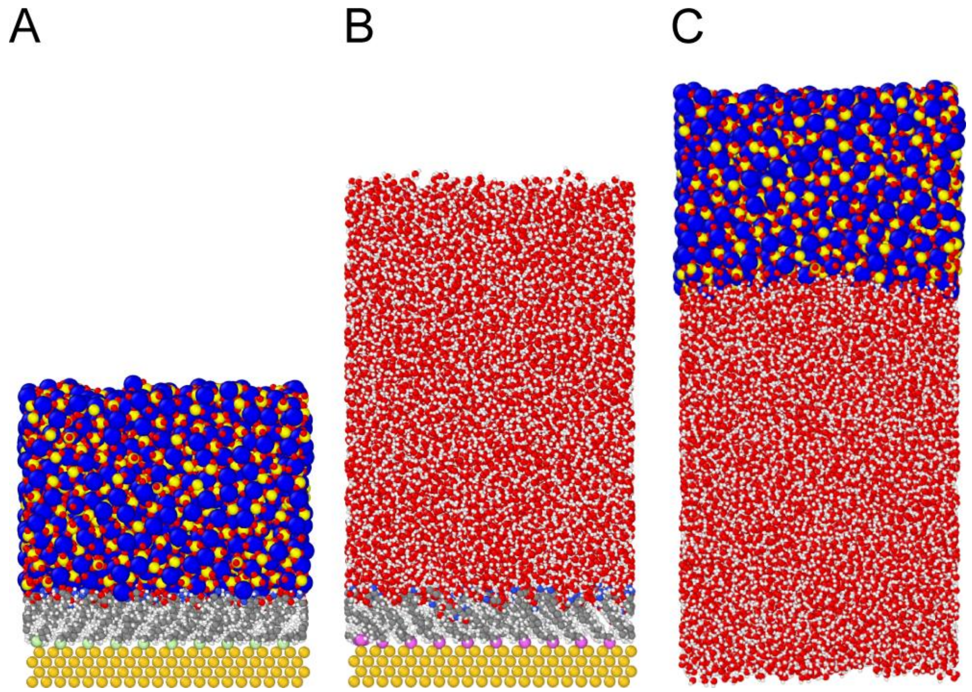

Supplementary Fig. 10 | The molecular models of calculating the interaction energy of (A) $CaSO_4$/surface, (B) water/surface, and (C) $CaSO_4$/water interfaces.

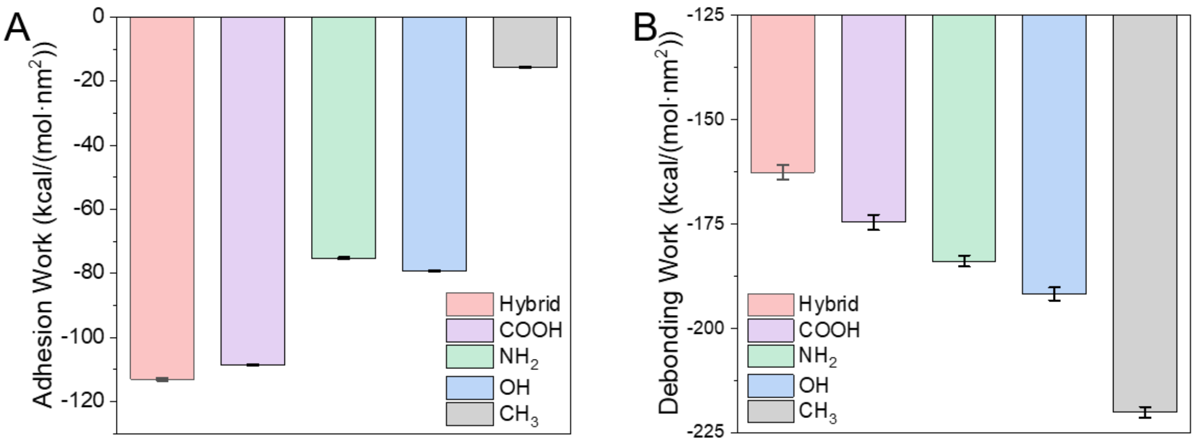

Supplementary Fig. 11 | (A) The adhesion work of the $CaSO_4$-surface interface. (B) The debonding work of water. The debonding work of water can be calculated using the following equation:

$$W_{debonding\_water}=(\Delta E_{water/surface}+\Delta E_{CaSO_4/water}-\Delta E_{CaSO_4/surface})/A$$

where $W_{debonding\_water}$, $\Delta E_{water/surface}$, $\Delta E_{CaSO4/water}$, $\Delta E_{CaSO4/surface}$, and A represent the debonding work of water, the interaction energy between water and the surface, the interaction energy between $CaSO_4$ and water, the interaction energy between $CaSO_4$ and the surface, and the contact area of the interface, respectively. Error bars are derived from the standard deviation of the average values calculated over the last 5 ns.

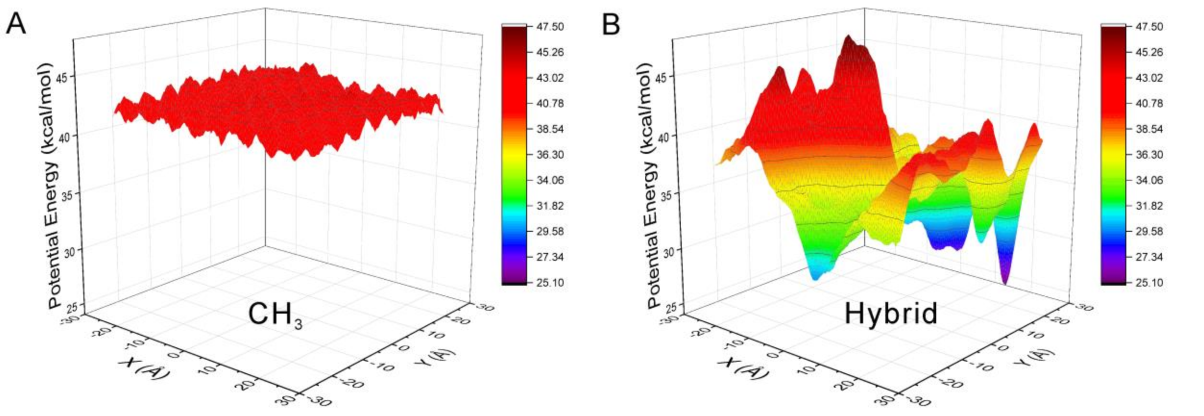

Supplementary Fig. 12 | The potential energy of surfaces terminated with (A) $-CH_3$ and (B) hybrid functional groups.

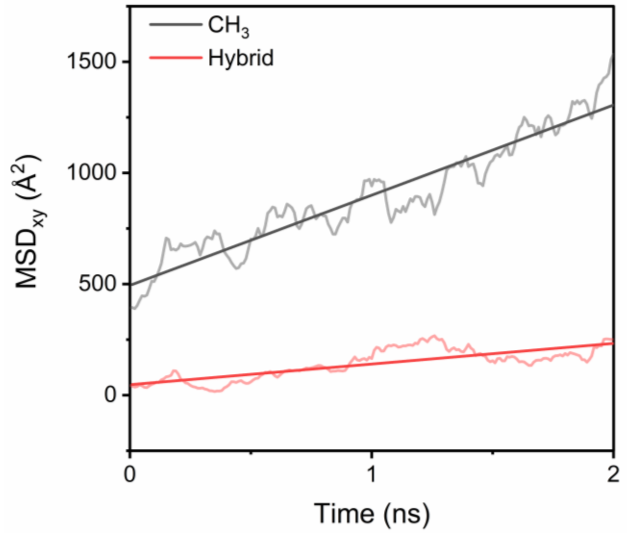

Supplementary Fig. 13 | The mean square displacement in the horizontal directions (MSD_{xy}) of $CaSO_4$ pairs above $-CH_3$ and hybrid functional group surfaces. The solid lines are the linear fittings of MSD_{xy} with time.

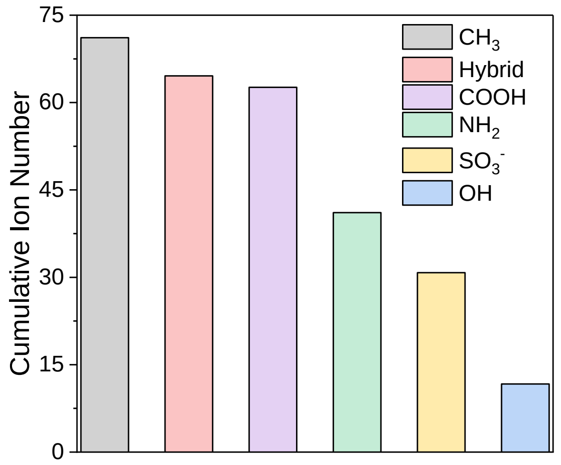

Supplementary Fig. 14 | The cumulative ion number within 25 Å along the perpendicular direction to the substrates.

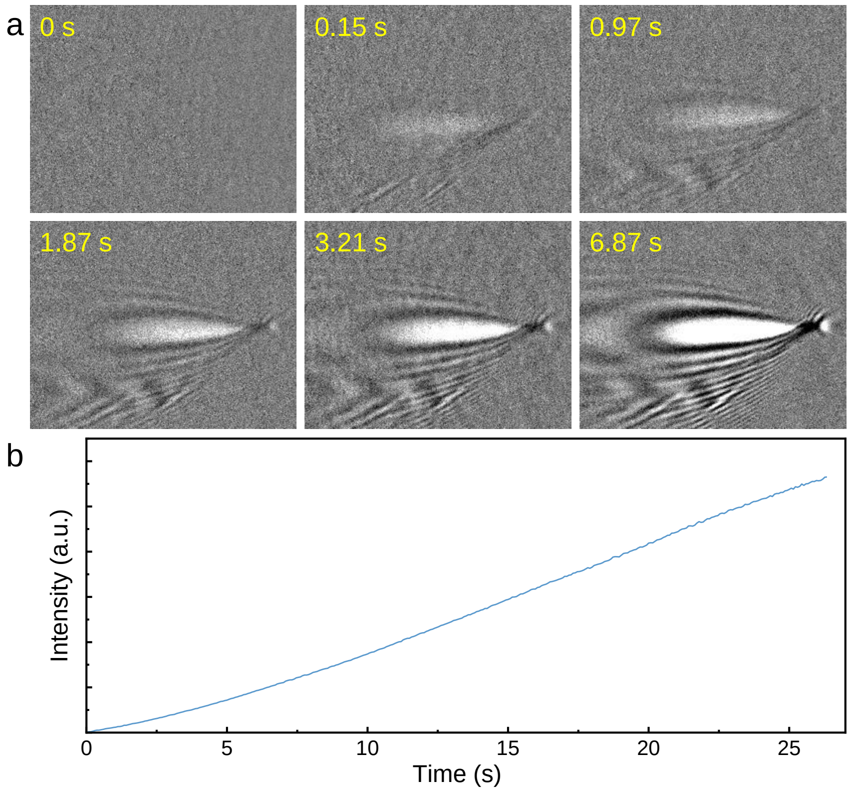

Supplementary Fig. 15 | (a) Snapshot plasmonic images and (b) intensity tracking of $CaSO_4$ nuclei during the nucleation process. The snapshot when the signal begins to appear was marked as the first frame (T = 0 s). Conditions: The plasmonic imaging system utilized a commercial inverted microscope (Ti microscope, Nikon Co., Japan) with a 100× oil-immersion objective lens (NA = 1.49). Illumination was provided by a 660 nm superluminescent diode to excite surface plasmon resonance. Standard glass slides were replaced with sensing chips modified by self-assembled monolayer with COOH groups. Plasmonic images were recorded by a CCD camera (Pike-032B, Allied Vision Technologies, USA) through a 0.46 × zoom-out lens (Nikon Co., Japan).

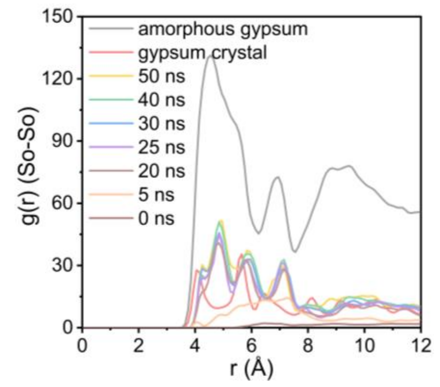

Supplementary Fig. 16 | The radial distribution function, g(r), of sulfur atom pairs of sulfate with time. $CaSO_4$ crystal and amorphous phase $CaSO_4$ were calculated for comparison.

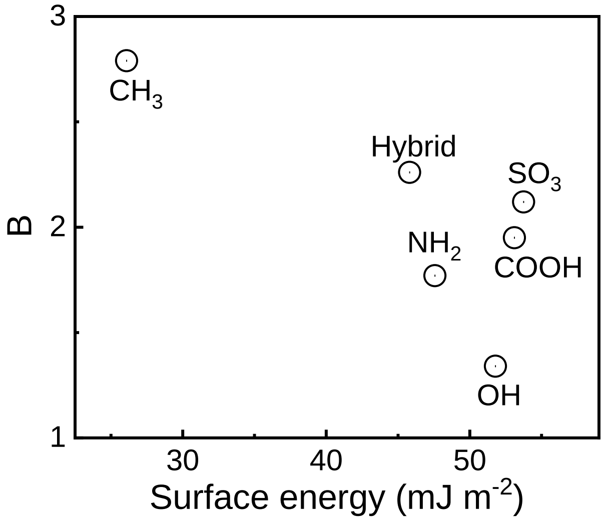

Supplementary Fig. 17 | Relating the extent of gypsum scaling (expressed as *B* value, Eqs. 6 and 7) to surface energy for heterogeneous nucleation derived from DLVO theory. Details on calculation methods and results are given in Supplementary Note1 and Supplementary Table 3.

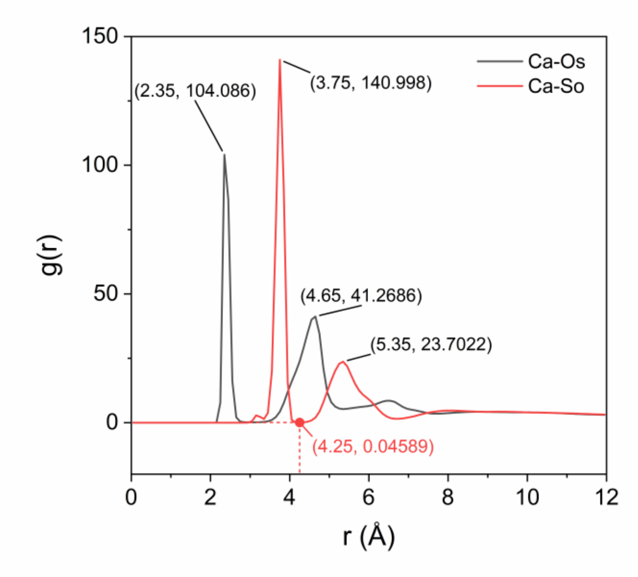

Supplementary Fig. 18 | The radial distribution function, g(r), of calcium atoms with oxygen and sulfur atoms.

Supplementary Table 1 | Recipes of feed solution for nucleation rate experiments.

Constituents				
$CaCl_2$ (mM)	40	50	60	70
Na_2SO_4 (mM)	40	50	60	70
Saturation index (σ)	0.97	1.24	1.43	1.63

Supplementary Table 2 | Raman peaks assignment in Fig. S3B to the characteristic peaks of gypsum[3, 4].

Wavenumber (cm^{-1})	assignment
520	v_2 symmetric bending
1004	v_1 symmetric stretch
1137	v_3 asymmetric stretch
3406	v_1 H_2O
3491	v_3 H_2O

Supplementary Table 3 | Surface energy of substrates with different functional groups calculated from contact angle measurements of three probe liquids (i.e., water, formamide, and bromonaphthalene).

Substrate	γ^{LW} (mJ m^{-2})	γ^{AB} (mJ m^{-2})	γ^{TOT} (mJ m^{-2})
$-CH_3$	25.28	0.81	26.09
−COOH	42.96	10.79	53.76
$-SO_3$	41.08	12.03	53.11
−OH	42.48	9.31	51.79
$-NH_2$	43.69	3.88	47.57
hybrid	43.62	2.18	45.81

Supplementary References

1. Gindl, M., Sinn, G., Gindl, W., Reiterer, A. & Tschegg, S. A comparison of different methods to calculate the surface free energy of wood using contact angle measurements. *Colloids Surf. A Physicochem. Eng. Asp.* **181**, 279-287 (2001).
2. Van Oss, C. Acid—base interfacial interactions in aqueous media. *Colloids Surf. A Physicochem. Eng. Asp.* **78**, 1-49 (1993).
3. Huang, W. et al. Mechanism of water extraction from gypsum rock by desert colonizing microorganisms. *Proc. Natl. Acad. Sci. USA* **117**, 10681-10687 (2020).
4. Zhang, Y. & Xue, D. In-situ micro-Raman spectroscopy study of gypsum crystallization driven by chemical reaction. *J. Mol. Struct.* **1210**, 128043 (2020).
